# Joining the Dots: Linking Disconnected Networks of Evidence Using Dose-Response Model-Based Network Meta-Analysis

**DOI:** 10.1177/0272989X20983315

**Published:** 2021-01-15

**Authors:** Hugo Pedder, Sofia Dias, Meg Bennetts, Martin Boucher, Nicky J. Welton

**Affiliations:** Department of Population Health Sciences, Bristol Medical School, University of Bristol, Bristol, UK; Centre for Reviews and Dissemination, University of York, York, North Yorkshire, UK; Pharmacometrics, Pfizer Ltd, Sandwich, Kent, UK; Pharmacometrics, Pfizer Ltd, Sandwich, Kent, UK; Department of Population Health Sciences, Bristol Medical School, University of Bristol, Bristol, UK

**Keywords:** disconnected, dose, MBNMA, meta-analysis, network, NMA, synthesis

## Abstract

**Background:**

Network meta-analysis (NMA) synthesizes direct and indirect evidence on multiple treatments to estimate their relative effectiveness. However, comparisons between disconnected treatments are not possible without making strong assumptions. When studies including multiple doses of the same drug are available, model-based NMA (MBNMA) presents a novel solution to this problem by modeling a parametric dose-response relationship within an NMA framework. In this article, we illustrate several scenarios in which dose-response MBNMA can connect and strengthen evidence networks.

**Methods:**

We created illustrative data sets by removing studies or treatments from an NMA of triptans for migraine relief. We fitted MBNMA models with different dose-response relationships. For connected networks, we compared MBNMA estimates with NMA estimates. For disconnected networks, we compared MBNMA estimates with NMA estimates from an “augmented” network connected by adding studies or treatments back into the data set.

**Results:**

In connected networks, relative effect estimates from MBNMA were more precise than those from NMA models (ratio of posterior SDs NMA v. MBNMA: median = 1.13; range = 1.04–1.68). In disconnected networks, MBNMA provided estimates for all treatments where NMA could not and were consistent with NMA estimates from augmented networks for 15 of 18 data sets. In the remaining 3 of 18 data sets, a more complex dose-response relationship was required than could be fitted with the available evidence.

**Conclusions:**

Where information on multiple doses is available, MBNMA can connect disconnected networks and increase precision while making less strong assumptions than alternative approaches. MBNMA relies on correct specification of the dose-response relationship, which requires sufficient data at different doses to allow reliable estimation. We recommend that systematic reviews for NMA search for and include evidence (including phase II trials) on multiple doses of agents where available.

Health care policy decisions increasingly use cost-effectiveness analysis to support decision making by health care professionals, a key element of which involves estimating the relative clinical effectiveness of multiple treatment options. This is typically done using network meta-analysis (NMA), which pools the results of randomized controlled trials (RCTs), enabling a comparison of multiple treatments simultaneously, provided they form a connected network of treatment comparisons.^1,2^ A connected network is one in which there is a path of RCT comparisons that can be followed between any pair of treatments in the network. For example, Figure 1a illustrates a connected network, whereas Figure 1b illustrates a network where treatments A and X are not connected to treatments B and Y. It is not possible to obtain a relative effect estimate for pairs of treatments that are not connected, for example B versus A in the network in Figure 1b, using standard NMA methods.

**Figure 1:**
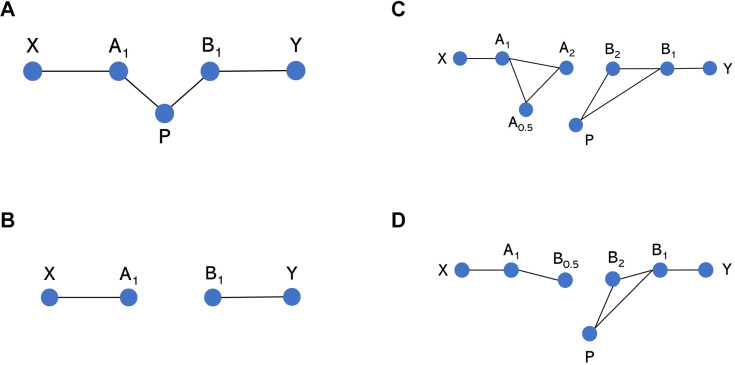
Network diagrams of potential network structures. Each node represents a different treatment, and each solid connecting line represents a head-to-head comparison for which evidence is available in a data set. A and B represent agents, whereas P represents placebo (equivalent to dose = 0 for any agent in the model-based network meta-analysis [MBNMA] modeling framework). Subscript numbers represent hypothetical doses. X and Y represent clustered “subnetworks” of treatments, which could be of any size but are only connected to other shown treatments via A and B, respectively. (a) All treatments are connected, and NMA can be used to estimate relative effects between any treatments. (b) Placebo data connecting treatments A and B are missing, meaning that they are disconnected and relative effects cannot be estimated for them or for any treatments in X versus Y. (c, d) Relative effects between A and B at any doses (or subsequently between treatments in X v. Y) cannot be estimated using NMA as they are not connected, but they can be estimated by using MBNMA to model the dose-response relationship.

In health technology assessment (HTA) it is common for networks of evidence to be disconnected or weakly connected, so that relative effects are either not estimable or very imprecisely estimated. This is in part due to new drugs obtaining marketing authorization before mature phase III RCT evidence has become available, partly because of the different comparator treatments being needed for marketing approval than by reimbursement agencies and also because of drugs being marketed in precisely defined patient populations, limiting the available evidence on comparator treatments.^3,4^

Various methods have been proposed to deal with disconnected networks in NMA.^5^ These include using observational or registry data,^6^ evidence in other populations,^7^ expert opinion,^8–10^ population adjustment methods,^11–13^ hierarchical models,^14^ and modeling intervention components^15,16^ to connect networks. For example, in an HTA comparing treatments for plaque psoriasis in children and young people,^7^ adalimumab was disconnected from the network, and evidence from an adult trial was used to enable an NMA comparing the treatments of interest. In another HTA on follicular lymphoma, different therapies with or without rituximab were compared, resulting in no common comparators^17^; however, by assuming the effects of the components in the combination therapies to be additive (with no interactions), the effects of the therapy given in both arms “cancels out,” so that each trial provides information on rituximab as an adjunct versus no adjunct, and the network connects.

A third example is an HTA for relapsed and refractory multiple myeloma,^18^ where there was no RCT evidence connecting pomalidomide with comparators panobinostat or bendamustine. Analysis of individual patient data from single arms and population adjustment methods were used to connect the network. However, all of these methods make strong and typically untestable assumptions.

Model-based network meta-analysis (MBNMA) is a new methodology that has the potential to connect networks of evidence in situations in which there is evidence on multiple doses of 1 or more agents, or observations at multiple follow-up times, by combining parametric models of dose response^19^ or time course^20^ with NMA in a statistically robust way that preserves randomization in included RCTs. One advantage of this approach is that it allows inclusion of trials from earlier phases of drug development into the network so that evidence on agents at unlicensed doses, or evidence at a variety of time points can be used to strengthen the evidence on the licensed treatments and time points that are of interest. For example, in the plaque psoriasis example,^7^ phase II dose-response information may be available on children for each treatment, which could connect the network without needing to rely on evidence in a different population (adults). Similarly, for the multiple myeloma example,^18^ there was evidence on multiple doses of bendamustine, which could potentially connect the network. Subsequent appraisals of newer drugs for multiple myeloma have compared multiple doses.^21^

Figures 1c and 1d illustrate 2 scenarios in which there are studies of A_1_ versus X and B_1_ versus Y (where treatments are defined by agent, A, B, X, Y, with subscript indicating dose, where dose = 1 is the licensed dose). A_1_ and B_1_ are disconnected, but there is evidence for a range of doses for at least 1 of the agents. In Figure 1c, by explicitly modeling the dose-response relationship using MBNMA, a placebo response (i.e., at dose = 0, where A_0_ = B_0_) is estimated for both agents (even agent A, for which a placebo has not been included in any trial). This connects the network, and a relative effect estimate between A_1_ and B_1_ can be obtained. In Figure 1d, A_1_ is connected only to B at a suboptimal dose and is not connected to placebo. However, by using MBNMA to model the dose-response relationship, B_0.5_ can be connected to other doses of B by interpolation, thus connecting the network and allowing for a comparison of A_1_ versus B_1_.

In this article, we aim to illustrate the potential of dose-response MBNMA to connect and strengthen evidence networks in a range of different scenarios. We begin by describing the MBNMA method.^19^ We then introduce a network of triptans for migraine relief and describe how we manipulate this data set to obtain a set of scenario networks with different features with which to illustrate the performance of the MBNMA method. We then present and compare results from MBNMA and NMA of the scenarios and end with a discussion.

## Methods

We first describe standard NMA, then the extension to dose-response MBNMA, and then how we generated a range of scenarios from the triptans data sets on which the methods are illustrated.

### Network Meta-Analysis

Following the methods of Lu and Ades,^1^ we define NMA as follows. For each study i , the aggregated data for arm k provides information on some parameter $\theta_{i,k}$ (e.g., probability, mean outcome), which is modeled using a generalized linear model^22^:

(1) $$g(\theta_{i,k})=\left\{ \begin{array}{c} \mu_{i}\;\mathrm{when}\;k=1\\ \;\mu_{i}+\delta_{i,k}\;\mathrm{when}\;k\geq 2\; \end{array}\right.$$

where g is a link function that transforms the outcome onto an appropriate scale (e.g., a logistic function for binary outcomes or an identity function for continuous outcomes), $\mu_{i}$ is the control arm (reference) treatment of study i , which is modeled as a nuisance parameter and given a vague prior, and $\delta_{i,k}$ is the study-specific relative treatment effect for the treatment used in arm k relative to the reference treatment in arm 1 of study i . In a random effects model, these are assumed to be normally distributed around a mean treatment effect that adheres to consistency relationships, with between-study variance $\tau^{2}$ that is common across treatment comparisons:

(2) $$\delta_{i,k}\sim N\left(d_{t_{i,k}}-d_{t_{i,1}},\tau^{2}\right)$$

where $d_{t_{i,k}}$ is the mean treatment effect of treatment $t_{i,k}$ compared with the network reference treatment. The consistency relationships reflect the comparison made between the treatment $t_{i,k}$ used on arm k and the treatment $t_{i,1}$ used on arm 1 of each study. A common effects model that assumes no between-study heterogeneity can be obtained by setting $\tau^{2}=0$ .

### Dose-Response MBNMA

The dose-response MBNMA model extends the standard NMA model to incorporate a dose-response relationship.^19^

We define a treatment in arm k of study i as a specific dose, $x_{i,k}$ , of a specific agent, $\;a_{i,k}$ . The model is exactly as for the NMA equation (1) above, but equation (2) is replaced with

(3) $$\delta_{i,k}\sim N(f(x_{i,k},a_{i,k})-f(x_{i,1},a_{i,1}),\tau^{2})$$

where $f(x_{i,k},a_{i,k})$ is a dose-response function for dose $x_{i,k}$ , agent $\;a_{i,k}$ , and $\tau^{2}$ is the between-study heterogeneity (set to zero for a common effects model). Multi-arm trials are dealt with in the same way as in standard NMA.^2^

Any dose-response function could be fitted, although this will be limited by the number of doses of an agent included in RCTs in the network. For example, for an exponential model,

$$f(x_{i,k},a_{i,k})=E_{0,i}+\beta_{a_{i,k}}(1-e^{-x_{i,k}})$$

where $E_{0,i}$ is the placebo response at $x_{i,k}=0$ in study i , and $\beta_{a_{i,k}}$ is the rate parameter for the agent in arm k of study i . The consistency equation in equation (3) means that the $E_{0,i}$ terms cancel out when forming the relative effects, so $E_{0,i}$ is not explicitly estimated within the model. In the exponential model, there is a single dose-response parameter to be estimated for each agent, meaning that studies with at least 2 doses (one of which could be placebo) of each agent are required to estimate $\beta_{a}$ .

Another commonly used dose-response model is the Emax function,^23^ which estimates the maximum response relative to placebo ( $E_{\max,a}$ ) and the dose at which half the maximum response can be achieved ( $ED_{50,a}$ ):

(4) $$f(x_{i,k},a_{i,k})=E_{0,i}+\frac{E_{\max,a_{i,k}}x_{i,k}}{ED_{50,a_{i,k}}+x_{i,k}}$$

Again, we do not explicitly estimate $E_{0,i}$ , as these terms cancel out when equation (4) is inserted into equation (3). The $E_{\max,a}$ and $ED_{50,a}$ parameters may be correlated, and this correlation can be estimated by specifying a bivariate normal distribution with a Wishart prior on the covariance matrix (see the Analyses and Implementation section and equation [5]). This extends to models with more than 2 parameters, in which a multivariate normal distribution can be specified.

To estimate both parameters of the Emax function, studies with at least 3 doses of a specific agent are required.

### Example Data Sets

A data set of published RCTs for the efficacy of triptans in migraine relief^24^ was used to illustrate the analyses. The outcome measured was the proportion of patients who were headache free at 2 h. This data set contains 22 treatments, 7 agents, and a placebo and was investigated in 70 studies. Doses are standardized to multiples of each agent’s “common” dose.^24^

From this complete data set, we generated manipulated data sets by removing specific treatments and studies to represent several scenarios that might be found in practice to compare the performance of NMA and MBNMA methods. If only a single arm remained in a study after excluding treatments, then that study was excluded. Complete and manipulated data sets generated for all scenarios can be found in the Supplementary Materials.

#### Scenario 1: Connected network

In scenario 1, data sets illustrate the use of MBNMA in connected networks with different amounts of dose-response information. Comparisons of interest are at the common dose (dose = 1).

##### Scenario 1A

**Figure 2:**
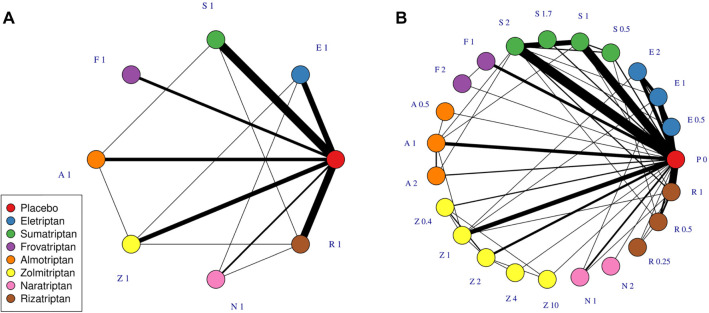
Network plots at the treatment level illustrating data sets in scenario 1A (A) and 1B (B). Each node represents a different treatment, and each solid connecting line represents a comparison for which evidence is available in the data set. The thickness of the connecting lines is proportional to the number of studies that compare the connected treatments. A treatment is defined as a specific dose of a specific agent. Treatments are named by the first letter of their agent and their dose, standardized to the common dose for each agent.

Scenario 1A is a manipulated data set composed of only a single common dose of each agent and placebo in the triptans data set (Figure 2A), which left 59 studies, 7 treatments (all common doses of different agents), and a placebo. This scenario may be similar to data sets found in HTAs or clinical guidelines, in which only comparisons between licensed doses of each agent are of interest and included in the evidence network.

##### Scenario 1B

Scenario 1B is the complete triptans data set including all doses and agents. This includes 70 studies, investigating 22 treatments, 7 agents, and a placebo (Figure 2B).

#### Scenarios 2 and 3: Disconnected networks

For simplicity, we suppose the objective is to compare 2 treatments of interest (agents of interest at the common dose). We take each pair of agents in turn and remove evidence on all other agents from the network, leaving only different doses of each agent of interest. These data sets are then manipulated further to obtain disconnected networks for scenarios 2 and 3 (see below). Manipulating the original data set in this way provides us with a number of different, simpler data sets that can be used to examine how the reliability of MBNMA changes depending on the agents and doses included.

We follow the approach taken by Beliveau et al.^25^ to compare MBNMA models fitted to disconnected networks with NMA models fitted to connected networks. We first fit MBNMA models to disconnected networks and calculated relative effects for the treatment comparison of interest in the network. Then we added in data to connect the networks, generating “augmented” data sets on which it was possible to fit NMA models. The relative effects calculated between the 2 sets of data were compared to assess the level of agreement.

#### Scenario 2: Disconnected due to absence of common comparator (e.g., placebo)

This illustrates a situation in which there is evidence on different doses for an agent of interest (e.g., from early-phase drug development trials) but there is no common comparator (Figure 1c).

To explore this, we generated a disconnected data set by removing all placebo arms from the data sets for each pair of agents (having already removed agents not of interest). For each of these networks, we also constructed an “augmented” data set by including comparisons between any doses of the included agents versus placebo so that the networks were fully connected and both MBNMA and NMA models could be fitted.

#### Scenario 3: Disconnected due to comparison with a dose that has not been evaluated in other trials

This illustrates a scenario shown in Figure 1d in which the treatment of interest (A_1_) has been investigated only in a study comparing a nonlicensed or nonoptimal dose of a comparator (B_0.5_) that is not connected to the dose of interest (B_1_) via any pathway of head-to-head evidence. In practice, this nonlicensed comparison might occur with a suboptimal dose of a comparator, such as in the GALLIUM trial comparing obinutuzumab for untreated advanced follicular lymphoma to rituximab administered for a shorter series of doses.^26^

Disconnected data sets were therefore generated such that studies comparing a common dose of one agent versus a nonoptimal dose of another were not connected to studies comparing other doses. Augmented data sets were then generated, which included comparisons between all doses of both agents, including the common dose, so that the networks were fully connected.

### Analyses and Implementation

All models were implemented using the package MBNMAdose version 0.2.7^27^ in R version 3.6.1 with a seed of 210489. Models were run until convergence was reached for all monitored parameters, as assessed by the Gelman-Rubin statistic^28^ and visual inspection of the chains.

The effective number of parameters were estimated using the plug-in method^29^ for NMAs and using the Kullback-Leibler divergence^30^ for MBNMAs. Deviance information criterion (DIC) was used to compare models, defined as the sum of the effective number of parameters added to the residual deviance.

Each data set was analyzed where possible using standard NMA and dose-response MBNMA. For both NMA and MBNMA, common and random effects models were compared. For MBNMA, a model selection strategy was used to determine a suitable model, in which first all models that were within 3 DIC points of the model with the lowest DIC were identified.^31^ Of these models, the simplest was preferred: models with common treatment effects were selected in preference to those with random treatment effects, and models with an exponential dose-response function were selected in preference to those with an Emax dose-response function.

This approach was used to allow selection of a dose-response function that could potentially explain as much heterogeneity as possible. Exponential and Emax were the only dose-response functions examined as there was a biological justification for their use over other possible functions (e.g., linear, quadratic).^23^

Vague normal prior distributions ( N(0,1000) ) were given to $d_{1,k}$ , $\mu_{i}$ , $\beta_{a_{i,k}}$ . For MBNMAs using the Emax function, a correlation was modeled between dose-response parameters by assigning them a multivariate normal prior:

(5) $$\left(\begin{array}{c} E_{\max,a_{i,k}}\\ \log\left(ED_{50,a_{i,k}}\right) \end{array}\right)\sim \mathrm{MVN}(0,\Sigma )$$

$ED_{50,a_{i,k}}$ was modeled on the log scale to ensure positive values. A minimally informative Wishart prior was used for $\Sigma^{-1}\sim \mathrm{Wishart}(\left(\begin{array}{cc} 1 & 0\\ 0 & 1 \end{array}\right),2)$ . The between-study SD, τ , was given a half-normal prior distribution ( N(0,400) ). Unless otherwise stated, results are presented as posterior medians and 95% credible intervals (95% CrIs).

## Results

### Scenario 1A

**Figure 3:**
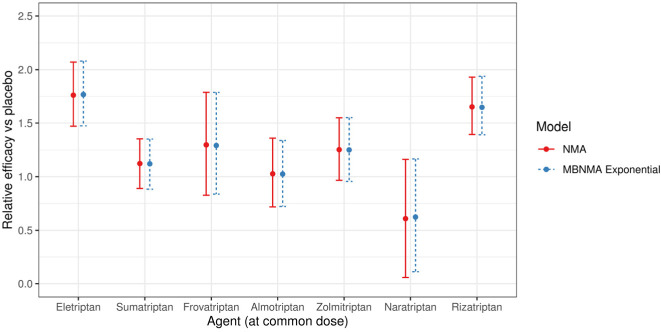
Forest plot showing the relative efficacy for each agent in scenario 1A at the common dose versus placebo, estimated from a common effects network meta-analysis (NMA) model and the selected common effects exponential model-based model. For each estimate, central points represent posterior medians and error bars represent 95% credible intervals.

In the network involving only licensed doses of each agent and placebo, it was only possible to fit an MBNMA model with a single parameter (i.e., linear or exponential models). Based on the exponential MBNMA model, relative effects estimated from selected NMA and MBNMA models were very similar (Figure 3). Between-study SD was reasonably high in both NMA (0.36; 95% CrI: 0.25, 0.50) and MBNMA (0.36; 95% CrI: 0.25, 0.50) models, and random effects models were selected in both instances. Model fit was similar for MBNMA and NMA models (Table 1). Because of the lack of dose-response information, there was no gain in precision of the estimates in the MBNMA model as compared with the NMA model.

**Table 1:** Model Fit Statistics for All Models Investigated in Scenario 1A and 1B Data Sets

Data Set	No. of Data Points	Residual Deviance	DIC^a^	pD^b^	Model	Dose-Response Function	Treatment Effects	Between-Study SD (95% CrI)
Scenario 1A	122	202.3	66.6	268.9	NMA	NA	Common	NA
Scenario 1A	122	124.0	96.3	220.3	NMA	NA	Random	0.36 (0.25, 0.50)
Scenario 1A	122	201.7	66.1	267.8	MBNMA	Exponential	Common	NA
Scenario 1A	122	124.0	96.2	220.2	MBNMA	Exponential	Random	0.36 (0.25, 0.50)
Scenario 1A	122	NC	NC	NC	MBNMA	Emax	Common	NA
Scenario 1A	122	NC	NC	NC	MBNMA	Emax	Random	NC
Scenario 1B	182	269.0	93.3	362.3	NMA	NA	Common	NA
Scenario 1B	182	190.6	131.6	322.2	NMA	NA	Random	0.27 (0.18, 0.37)
Scenario 1B	182	296.5	77.1	373.6	MBNMA	Exponential	Common	NA
Scenario 1B	182	189.4	125.1	314.5	MBNMA	Exponential	Random	0.28 (0.20, 0.37)
Scenario 1B	182	266.8	80.9	347.7	MBNMA	Emax	Common	NA
Scenario 1B	182	191.7	121.6	121.6	MBNMA	Emax	Random	0.24 (0.16, 0.34)

a DIC: deviance information criterion = pD + residual deviance.

b pD: The effective number of parameters calculated using the Kullback-Leibler divergence^30^ for model-based network meta-analysis (MBNMA) and the plugin method^29^ for NMA. NC, Markov chain Monte Carlo chains did not converge; model was not identifiable.

### Scenario 1B

In Scenario 1B, all available doses of each agent and placebo were included. Random effects models were selected for the NMA and MBNMA models. An Emax dose-response function was selected for the MBNMA model, with an estimated correlation between Emax and ED50 dose-response parameters of 0.57 (95% CrI −0.53, 0.93; Table 1).

**Figure 4:**
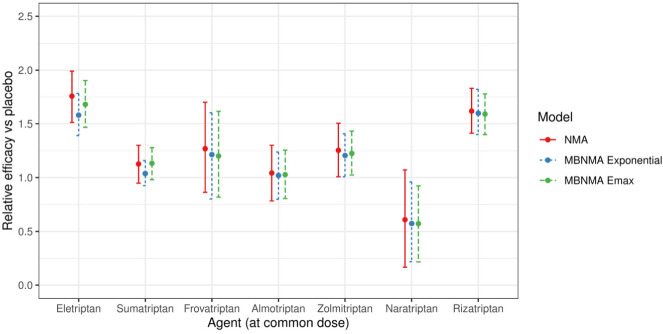
Forest plot showing the relative efficacy for each agent in scenario 1B at the common dose versus placebo, estimated from a random effects network meta-analysis (NMA) model and random effects exponential and Emax model-based NMA models. For each estimate, central points represent posterior medians and error bars represent 95% CrIs.

The relative effects from both NMA and MBNMA were more precise for all agents at the common dose than in scenario 1A because of the inclusion of trials comparing nonlicensed doses (Figure 4). Furthermore, MBNMA estimates were more precise than NMA estimates because of the additional information gained from modeling the dose-response relationship (Figure 4). The between-study SD was also slightly reduced for the MBNMA model (0.24; 95% CrI: 0.16, 0.34) as compared with the NMA model (0.27; 95% CrI: 0.18, 0.38).

### Scenario 2

It was possible to fit MBNMA models for 15 different agent versus agent comparisons generated in scenario 2 (Supplementary Figure S1), but this was not possible for agent pairs that included naratriptan because removing the placebo arms left only single arms of studies including naratriptan.

In all disconnected data sets, an exponential dose-response MBNMA was selected with common treatment effects (Table 2). NMA models could not be estimated because of the networks being disconnected.

**Table 2:** Model Fit Statistics for Selected Models in Each Data Set Analyzed in Scenario 2

Data Set Number	Data Set	Agent 1	Agent 2	No. of Data Points	Residual Deviance	DIC^a^	pD^b^	Model	Dose-Response Function	Treatment Effects	Between-Study SD
1	Initial	Almotriptan	Rizatriptan	13	12.0	20.0	8.0	MBNMA	Exponential	Common	NA
1	Augmented	Almotriptan	Rizatriptan	45	48.9	81.5	32.6	MBNMA	Exponential	Random	0.27 (0.09−0.5)
1	Augmented	Almotriptan	Rizatriptan	45	48.1	83.3	35.3	NMA	NA	Random	0.32 (0.12−0.59)
2	Initial	Almotriptan	Zolmitriptan	14	11.0	19.2	8.2	MBNMA	Exponential	Common	NA
2	Augmented	Almotriptan	Zolmitriptan	44	42.1	63.0	21.0	MBNMA	Emax	Common	NA
2	Augmented	Almotriptan	Zolmitriptan	44	45.3	71.6	26.3	NMA	NA	Common	NA
3	Initial	Eletriptan	Almotriptan	22	24.0	36.3	12.3	MBNMA	Exponential	Common	NA
3	Augmented	Eletriptan	Almotriptan	46	48.1	81.7	33.6	MBNMA	Emax	Random	0.3 (0.14−0.5)
3	Augmented	Eletriptan	Almotriptan	46	48.3	84.5	36.3	NMA	NA	Random	0.34 (0.16−0.58)
4	Initial	Eletriptan	Frovatriptan	18	21.6	31.9	10.2	MBNMA	Exponential	Common	NA
4	Augmented	Eletriptan	Frovatriptan	42	42.1	76.0	34.0	MBNMA	Emax	Random	0.4 (0.23−0.67)
4	Augmented	Eletriptan	Frovatriptan	42	42.8	77.0	34.2	NMA	NA	Random	0.43 (0.23−0.71)
5	Initial	Eletriptan	Rizatriptan	23	27.9	39.9	12.1	MBNMA	Exponential	Common	NA
5	Augmented	Eletriptan	Rizatriptan	61	63.1	110.2	47.2	MBNMA	Emax	Random	0.38 (0.23−0.57)
5	Augmented	Eletriptan	Rizatriptan	61	63.9	112.0	48.1	NMA	NA	Random	0.4 (0.24−0.63)
6	Initial	Eletriptan	Sumatriptan	36	42.7	60.8	18.1	MBNMA	Exponential	Common	NA
6	Augmented	Eletriptan	Sumatriptan	90	92.3	157.0	64.7	MBNMA	Emax	Random	0.31 (0.19−0.44)
6	Augmented	Eletriptan	Sumatriptan	90	92.4	158.3	65.9	NMA	NA	Random	0.31 (0.19−0.45)
7	Initial	Eletriptan	Zolmitriptan	22	24.4	35.3	10.9	MBNMA	Exponential	Common	NA
7	Augmented	Eletriptan	Zolmitriptan	57	57.6	98.9	41.3	MBNMA	Emax	Random	0.29 (0.13−0.48)
7	Augmented	Eletriptan	Zolmitriptan	57	59.1	102.1	43.0	NMA	NA	Random	0.32 (0.15−0.53)
8	Initial	Frovatriptan	Almotriptan	8	5.8	12.1	6.3	MBNMA	Exponential	Common	NA
8	Augmented	Frovatriptan	Almotriptan	26	31.6	44.7	13.1	MBNMA	Exponential	Common	NA
8	Augmented	Frovatriptan	Almotriptan	26	34.7	50.8	16.2	NMA	NA	Common	NA
9	Initial	Frovatriptan	Rizatriptan	9	9.7	16.1	6.5	MBNMA	Exponential	Common	NA
9	Augmented	Frovatriptan	Rizatriptan	41	42.3	74.8	32.5	MBNMA	Emax	Random	0.41 (0.21−0.71)
9	Augmented	Frovatriptan	Rizatriptan	41	42.4	76.0	33.5	NMA	NA	Random	0.45 (0.22−0.8)
10	Initial	Frovatriptan	Zolmitriptan	10	8.6	14.3	5.7	MBNMA	Exponential	Common	NA
10	Augmented	Frovatriptan	Zolmitriptan	38	44.4	62.9	18.5	MBNMA	Emax	Common	NA
10	Augmented	Frovatriptan	Zolmitriptan	38	47.2	70.5	23.3	NMA	NA	Common	NA
11	Initial	Sumatriptan	Almotriptan	24	25.8	39.0	13.2	MBNMA	Exponential	Common	NA
11	Augmented	Sumatriptan	Almotriptan	77	78.4	130.3	51.9	MBNMA	Emax	Random	0.25 (0.13−0.39)
11	Augmented	Sumatriptan	Almotriptan	77	79.2	134.2	55.1	NMA	NA	Random	0.25 (0.1−0.41)
12	Initial	Sumatriptan	Frovatriptan	22	24.6	36.3	11.7	MBNMA	Exponential	Common	NA
12	Augmented	Sumatriptan	Frovatriptan	72	71.9	123.2	51.4	MBNMA	Exponential	Random	0.32 (0.18−0.48)
12	Augmented	Sumatriptan	Frovatriptan	72	72.2	126.2	54.0	NMA	NA	Random	0.32 (0.17−0.49)
13	Initial	Sumatriptan	Rizatriptan	25	28.7	41.4	12.7	MBNMA	Exponential	Common	NA
13	Augmented	Sumatriptan	Rizatriptan	93	96.7	163.5	66.8	MBNMA	Emax	Random	0.3 (0.18−0.43)
13	Augmented	Sumatriptan	Rizatriptan	93	96.6	165.0	68.4	NMA	NA	Random	0.3 (0.18−0.44)
14	Initial	Sumatriptan	Zolmitriptan	28	29.9	43.7	13.9	MBNMA	Exponential	Common	NA
14	Augmented	Sumatriptan	Zolmitriptan	88	88.0	150.0	61.9	MBNMA	Emax	Random	0.26 (0.12−0.4)
14	Augmented	Sumatriptan	Zolmitriptan	88	89.1	151.6	62.5	NMA	NA	Random	0.26 (0.09−0.41)
15	Initial	Zolmitriptan	Rizatriptan	15	14.9	22.9	8.0	MBNMA	Exponential	Common	NA
15	Augmented	Zolmitriptan	Rizatriptan	56	58.9	95.0	36.1	MBNMA	Emax	Random	0.26 (0.07−0.47)
15	Augmented	Zolmitriptan	Rizatriptan	56	58.8	100.7	41.9	NMA	NA	Random	0.3 (0.11−0.55)

a DIC: deviance information criterion = pD + residual deviance.

b pD: The effective number of parameters calculated using the Kullback-Leibler divergence^30^ for model-based network analysis (MBNMA) and the plugin method^29^ for NMA.

**Figure 5:**
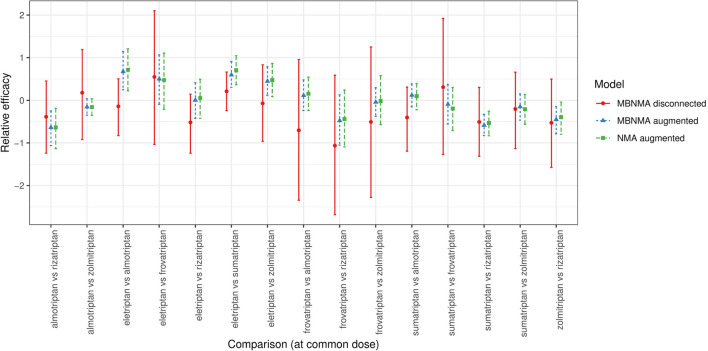
Forest plot showing the relative efficacies between focal treatments (two agents at their common dose) in each distinct data set generated for scenario 2 (see Supplementary Figures S1 and S2), estimated from selected NMA and MBNMA models in disconnected and augmented data sets. Relative effects cannot be estimated in NMA models for the disconnected data sets because treatments are not connected via pathways of head-to-head evidence. For each estimate, the central points represent posterior medians, and error bars represent 95% CrIs.

Relative effects estimated using MBNMA had high uncertainty (Figure 5), reflecting both the sparsity of data in the networks (number of data points per data set: median = 22; range = 8 to 36) and the fact that no placebo evidence was available with which to inform the dose-response relationship at lower doses.

Augmenting the data sets by adding in placebo arms to connect the network enabled NMA models to be estimated. For MBNMA models, an Emax dose-response function was selected for 12 of 15 data sets. Random treatment effects were selected over common effects in 12 of 15 data sets for both NMA and MBNMA models.

For most comparisons, results in the disconnected data sets were consistent with those in augmented data sets (Figure 5). However, for comparisons of almotriptan, rizatriptan, and sumatriptan with eletriptan, estimates from the disconnected data sets were further away from the posterior medians of augmented data set estimates, and results were less consistent.

Within augmented data sets, MBNMA estimates were very similar to corresponding NMA estimates but with slightly increased precision leading to narrower 95% CrIs, which were typically within those of the NMA estimates. The ratio of posterior SDs for the NMA estimates compared with the MBNMA estimates for each comparison had a median of 1.13 (range, 1.04 to 1.68).

### Scenario 3

Given the constraints of the original triptans data set, we were only able to generate suitable manipulated data sets for this scenario using higher doses of sumatriptan than the common dose. We were able to construct 3 networks to illustrate this scenario. Disconnected data sets therefore included a study comparing a common dose of one agent (either almotriptan/eletriptan/rizatriptan) versus twice the common dose of sumatriptan that was disconnected from studies comparing other doses of sumatriptan (including placebo; Supplementary Figure S3). Augmented data sets were similar but included comparisons between the common dose of almotriptan/eletriptan/rizatriptan and all doses of sumatriptan so that the network was fully connected (Supplementary Figure S4).

For all data sets generated in scenario 3, exponential MBNMA models with random treatment effects were selected (Table 3). NMA models could not be estimated because the networks were disconnected. Precision was typically higher in relative effects for data sets generated in scenario 3 than in scenario 2, although it is unclear whether this was due to the specific inclusion of placebo within the data set or due to the increased evidence available in scenario 3 (Tables 2 and 3). When augmenting the data sets to enable estimation of NMA models, random effects models were selected in all data sets for both NMA and MBNMA models.

**Table 3:** Model Fit Statistics for Selected MBNMA and NMA Models in Each Data Set Analyzed in Scenario 3

Dataset Number	Data Set	Agent 1	Agent 2	No. of Data Points	Residual Deviance	DIC^a^	pD^b^	Model	Dose-Response Function	Treatment Effects	Between-Study SD
1	Initial	Almotriptan	Sumatriptan	38	37.5	66.6	29.1	MBNMA	Exponential	Random	0.30 (0.10−0.54)
1	Augmented	Almotriptan	Sumatriptan	74	74.3	127.5	53.3	MBNMA	Exponential	Random	0.28 (0.16−0.44)
1	Augmented	Almotriptan	Sumatriptan	74	75.3	128.6	53.3	NMA	NA	Random	0.27 (0.12−0.42)
2	Initial	Eletriptan	Sumatriptan	38	37.2	66.8	29.6	MBNMA	Exponential	Random	0.29 (0.11−0.54)
2	Augmented	Eletriptan	Sumatriptan	80	81.1	141.0	59.8	MBNMA	Exponential	Random	0.35 (0.22−0.52)
2	Augmented	Eletriptan	Sumatriptan	80	81.0	142.5	61.6	NMA	NA	Random	0.36 (0.22−0.53)
3	Initial	Rizatriptan	Sumatriptan	40	38.6	69.2	30.6	MBNMA	Exponential	Random	0.28 (0.11−0.53)
3	Augmented	Rizatriptan	Sumatriptan	87	89.0	152.9	63.9	MBNMA	Exponential	Random	0.32 (0.21−0.48)
3	Augmented	Rizatriptan	Sumatriptan	87	89.5	154.1	64.6	NMA	NA	Random	0.32 (0.20−0.47)

a DIC: deviance information criterion = pD + residual deviance.

b pD: The effective number of parameters calculated using the Kullback-Leibler divergence^30^ for model-based network meta-analysis (MBNMA) and the plugin method^29^ for NMA.

**Figure 6:**
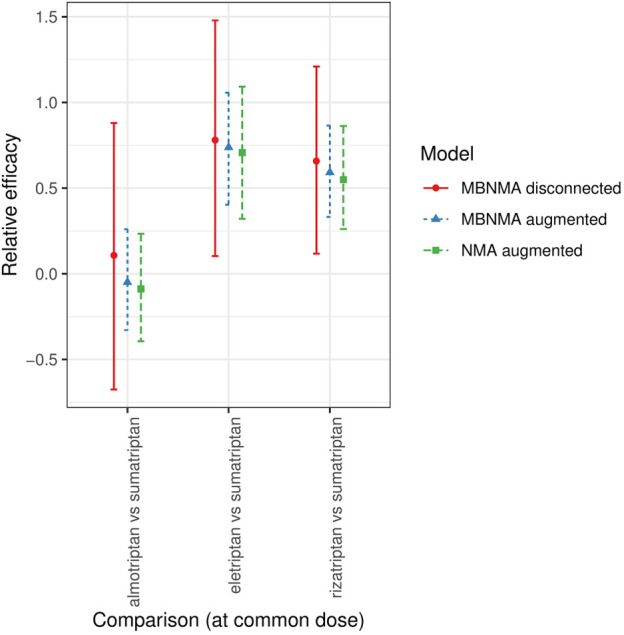
Forest plot showing the relative efficacies between focal treatments (2 agents at their common dose) in each distinct data set generated for scenario 3 (see Supplementary Figure S3 and S4), estimated from selected network meta-analysis (NMA) and model-based NMA (MBNMA) models in disconnected and augmented data sets. Relative effects cannot be estimated in NMA models for the disconnected data sets because treatments are not connected via pathways of head-to-head evidence. For each estimate, the central points represent posterior medians and error bars represent 95% credible intervals.

For all three comparisons, relative effects (either from MBNMA or NMA) in augmented data sets were entirely within the 95% CrIs of those estimated from MBNMAs in the disconnected data sets (Figure 6), suggesting that results were in agreement.

For augmented data sets, MBNMA estimates were very similar to NMA estimates. There was slightly increased precision in MBNMA estimates, leading to narrower 95% CrIs. The ratios of the posterior SDs for the NMA estimates compared with the corresponding MBNMA estimates for each comparison at the common dose were 1.03, 1.16, and 1.13 for almotriptan, eletriptan, and rizatriptan, respectively, versus sumatriptan.

## Discussion

This study illustrates several scenarios in which dose-response MBNMA can add value as compared with standard NMA methods, either by improving precision or by connecting networks to enable comparisons between treatments of interest to be made. Connecting and strengthening networks is enabled by including additional evidence on nonoptimal doses and via the modeling of a functional dose-response relationship, which can act as a link between disconnected treatments, either between different doses of the same agent along the dose-response curve or between different agents via extrapolation of the placebo response.

Evidence on nonlicensed doses is not typically included in HTA submissions; however, such evidence will often exist and, if included using MBNMA, could add value by increasing precision even in connected networks. HTAs where multiple doses are of interest could also benefit from modeling using MBNMA. Examples include treatments for moderate-to-severe plaque psoriasis^32–34^ and retigabine for the adjunctive treatment of partial-onset seizures in epilepsy.^35^

In scenarios in which the networks were disconnected (scenarios 2 and 3), we found that MBNMA allowed estimation of relative effects, which were consistent with NMA estimates obtained in augmented data sets where connections were added back into the network.

In the situation in which dose-response information is available on 2 agents but there is no direct comparison connecting the agents (scenario 2), we found that, although MBNMA models could be estimated, there was limited information with which to estimate a complex dose-response function because of the comparatively few different doses of each agent in the triptans data set, particularly at lower doses, when there is no placebo information. This was more problematic for eletriptan, as the dose-response relationship was better described by an Emax than an exponential function, which resulted in relative effects that were typically lower as compared with those estimated from augmented data sets for eletriptan versus several other agents. Although phase II studies would typically include a placebo arm, these studies may remain unpublished, so a manufacturer may have placebo evidence for their own agent but not necessarily for that of their competitors.

In the situation in which there was a direct comparison of the agents of interest but the network was disconnected because one of the agents was trialed at a nonoptimal dose (scenario 3), MBNMA was able to link agents at the optimal dose. Although there were only 3 possible combinations of agents in the triptans data set for which it was possible to examine this scenario, estimates from augmented and disconnected data sets were in agreement. The reliability of the results from this scenario were due to considerable information at different doses for the agent connected via the dose-response relationship (sumatriptan in all 3 data sets). It is unclear how frequently these evidence structures might arise in HTAs, as submissions typically compare only licensed doses.

### Comparison with Other Methods for Disconnected Networks

Dose-response MBNMA has several advantages as compared with other methods for linking disconnected networks provided sufficient data are available for estimation. In particular, the method uses only randomized evidence, and the statistical approach respects the randomization in RCTs. This means that the estimates are unbiased provided there are no differences in treatment effect modifiers between studies (the standard assumption made in NMA) and the dose-response function is not misspecified. The assumptions made regarding the dose-response relationship are also testable by evaluating the model’s fit. Furthermore, MBNMA can be fitted using aggregate data only, without the need for individual patient data.

MBNMA is distinct from model-based meta-analysis (MBMA), which models dose response but typically pools absolute rather than relative effects.^36–38^ MBMA can be used with disconnected networks and allows inclusion of single-arm studies. However, it can produce biased estimates because of differences between studies in prognostic factors, as it violates randomization by ignoring within-study comparisons.^39^

Another approach for dealing with disconnected networks is to fit a random effects model for the absolute effects on a specific reference treatment A. This random effects model is used to predict a treatment A effect in any study that is disconnected from the network, thus enabling that study to connect via treatment A.^14^ This method does not require individual patient data and can incorporate single-arm studies. However, it can introduce important bias because it breaks randomization by allowing within-study information to be influenced by information outside the study.^40^ It also relies on there being sufficient studies that include treatment A to enable estimation of the random effects model. If there is substantial heterogeneity between studies, then the predicted A effect in disconnected studies will be imprecisely estimated, and network connections will be tenuous. The model also assumes that the baseline model has been correctly specified, which may require adjusting for study-level factors that affect the baseline response.^8^ Beliveau et al.^25^ applied random baseline effect NMA models to disconnected networks, finding that there was generally good overlap between random baseline models and standard NMA models in subsets of 2 different data sets. However, White et al.^40^ showed that bias would occur if underlying studies had different baseline predictors,^40^ and it is not clear how frequently this might be the case in practice. There is also no way of testing the assumption that the baseline effect has been correctly specified, and important predictors may not be reported in included studies.

Population adjustment methods such as matched adjusted indirect comparisons^11,12^ or simulated treatment comparisons^13^ have also been used to link disconnected treatments. These methods predict an absolute effect of a disconnected treatment Y in the population of a trial including treatment X, and the prediction is analyzed as if it were an additional arm in the trial including X. However, the validity of comparisons relies on the assumption that the differences in absolute effects between studies can be fully explained by adjustment of prognostic variables (those that affect the outcome) as well as effect modifiers (those that alter the treatment effect).^41^ This is a very strong assumption that is impossible to test within the analysis, and it is unlikely that each trial has collected information on the same set of potential effect modifiers and prognostic factors. If this assumption does not hold, then the resulting relative effects between disconnected treatments will be biased.^41^ These methods also require individual patient data to be available for at least 1 RCT, although in HTA, this is typically available for the manufacturer’s trial.

An alternative method that makes use of functional assumptions regarding treatment definitions and can be performed using aggregate data is component network meta-analysis.^15,16^ This splits combinations of treatments into different components, allowing for networks to be connected if treatments in separate subnetworks share at least 1 common component,^42^ and it has been used for this purpose in an analysis of cognitive behavioral therapies for panic disorder.^43^

Although a network may be disconnected for a particular outcome, other correlated outcomes may be available, and a joint analysis using multivariate NMA may provide relative effect estimates between treatments that are disconnected for a given outcome, although correlations must be high to enable this.^44^ This approach was used to model the effects of first- and second-line therapies for rheumatoid arthritis.^45^

A more powerful approach is to model a structural relationship between multiple outcomes. Lu et al.^46^ used piecewise constant models to synthesize different networks (some of which were disconnected) at multiple follow-up times, and fractional polynomial models have also been used.^47^ Time-course MBNMA^20^ provides a general framework to fit a functional time-course relationship, which can connect networks and provide considerably more precision than modeling the correlation alone.^20^ Time-course MBNMA could have potential benefit in HTAs; for example, treatments for relapsing multiple sclerosis typically report at multiple time points, but economic models are based on 6-mo follow-up, which is not reported for all treatments.^48^

Assuming a common or exchangeable effect among similar treatments can be used as a way of connecting networks or dealing with sparse evidence structures,^49,50^ for example, drugs in the same class with a similar mechanism of action or biosimilar products. However, assuming a common effect is a very strong assumption that can be difficult to justify, and assuming exchangeable effects will shrink treatment effects toward a class mean effect, which may not be realistic.

Other approaches that have been proposed to connect networks include incorporating nonrandomized evidence^6^ or expert opinion^8–10^ to inform a prior distribution for the relative effect between the disconnected treatments. However, observational evidence is vulnerable to a range of biases, which may invalidate relative effect estimates, and although expert opinion may be useful to put some bounds on plausible effect sizes, it is subjective and prone to bias.

### Limitations

Although there are advantages of using dose-response MBNMA, there are also some clear limitations. The method is sensitive to misspecification of the dose-response function, and more complex dose-response models such as the Emax model require data on multiple doses of different agents to be able to estimate them. Doses that are more widely distributed will be more informative in identifying points of curvature in the dose-response function and are therefore likely to be important for mitigating bias.^51^ This is highlighted by the lack of placebo data in scenario 2, which generally resulted in underestimated relative effects for eletriptan versus other agents in disconnected data sets.

With only a single dose and placebo (or 2 doses without placebo) for each agent, only simple MBNMA models can be fitted, such as linear or exponential functions. Model fit statistics cannot help distinguish between models in this situation, although there may be some biological justification for an exponential function.^23^ External evidence may be helpful to support the choice of dose-response function, perhaps from data on related agents, or the same agents in different populations. Sharing either $ED_{50}$ or $E_{\max}$ across agents within a class may make the Emax model easier to fit when data are limited, although this should be done only if there is clinical justification. Simulation studies to explore the performance of MBNMA models for different evidence structures would be a useful area for further work.

## Conclusions

NMA relies on networks of treatments being connected. MBNMA allows reconnecting of networks via the dose-response relationship when evidence on multiple doses of agents is available. In our manipulated data sets, MBNMA estimates were in agreement with those from NMA, had connecting studies been available. MBNMA makes fewer assumptions than other methods for linking disconnected networks, with the only additional assumption over NMA being that the dose-response relationship is correctly specified. This assumption can be tested by examining the fit of the model to the data and/or based on the agent pharmacology. MBNMA can be performed using aggregate data and can add precision over NMA even in connected networks, when multiple doses are available.

MBNMA does, however, require information on multiple doses for each agent, particularly to estimate more complex dose-response functions. We therefore recommend that systematic reviews supporting HTA should broaden their scope to include all doses in instances in which the use of dose-response MBNMA is expected to be of value. We also urge manufacturers to publish their phase II study results, so that reimbursement decisions can make full use of the evidence available. Early-phase evidence is taken into consideration when gaining regulatory approval, and incorporating this information into HTA may help bridge the evidence gap between regulators and reimbursement bodies.^3,4^

## Research Data

sj-csv-1-mdm-10.1177_0272989X20983315 for Joining the Dots: Linking Disconnected Networks of Evidence Using Dose-Response Model-Based Network Meta-Analysis sj-csv-1-mdm-10.1177_0272989X20983315 for Joining the Dots: Linking Disconnected Networks of Evidence Using Dose-Response Model-Based Network Meta-Analysis by Hugo Pedder, Sofia Dias, Meg Bennetts, Martin Boucher and Nicky J. Welton in Medical Decision Making This article is distributed under the terms of the Creative Commons Attribution 4.0 License ( http://www.creativecommons.org/licenses/by/4.0/) which permits any use, reproduction and distribution of the work without further permission provided the original work is attributed as specified on the SAGE and Open Access pages ( https://us.sagepub.com/en-us/nam/open-access-at-sage).

sj-pdf-3-mdm-10.1177_0272989X20983315 – Supplemental material for Joining the Dots: Linking Disconnected Networks of Evidence Using Dose-Response Model-Based Network Meta-Analysis Supplemental material, sj-pdf-3-mdm-10.1177_0272989X20983315 for Joining the Dots: Linking Disconnected Networks of Evidence Using Dose-Response Model-Based Network Meta-Analysis by Hugo Pedder, Sofia Dias, Meg Bennetts, Martin Boucher and Nicky J. Welton in Medical Decision Making

sj-RData-2-mdm-10.1177_0272989X20983315 for Joining the Dots: Linking Disconnected Networks of Evidence Using Dose-Response Model-Based Network Meta-Analysis sj-RData-2-mdm-10.1177_0272989X20983315 for Joining the Dots: Linking Disconnected Networks of Evidence Using Dose-Response Model-Based Network Meta-Analysis by Hugo Pedder, Sofia Dias, Meg Bennetts, Martin Boucher and Nicky J. Welton in Medical Decision Making This article is distributed under the terms of the Creative Commons Attribution 4.0 License ( http://www.creativecommons.org/licenses/by/4.0/) which permits any use, reproduction and distribution of the work without further permission provided the original work is attributed as specified on the SAGE and Open Access pages ( https://us.sagepub.com/en-us/nam/open-access-at-sage).
