# Supplementary material for: Joining the Dots: Linking Disconnected Networks of Evidence Using Dose-Response Model-Based Network Meta-Analysis
Source: Med Decis Making. 2021 Jan 15;41(2):194–208. doi: 10.1177/0272989X20983315 (PMC7879230; doi:10.1177/0272989X20983315)
Supplement: sj-pdf-3-mdm-10.1177_0272989X20983315 – Supplemental material for Joining the Dots: Linking Disconnected Networks of Evidence Using Dose-Response Model-Based Network Meta-Analysis [file sj-pdf-3-mdm-10.1177_0272989X20983315.pdf]

Joining the Dots – Linking disconnected networks of evidence using dose-response Model-Based Network Meta-Analysis

Supplementary Figures

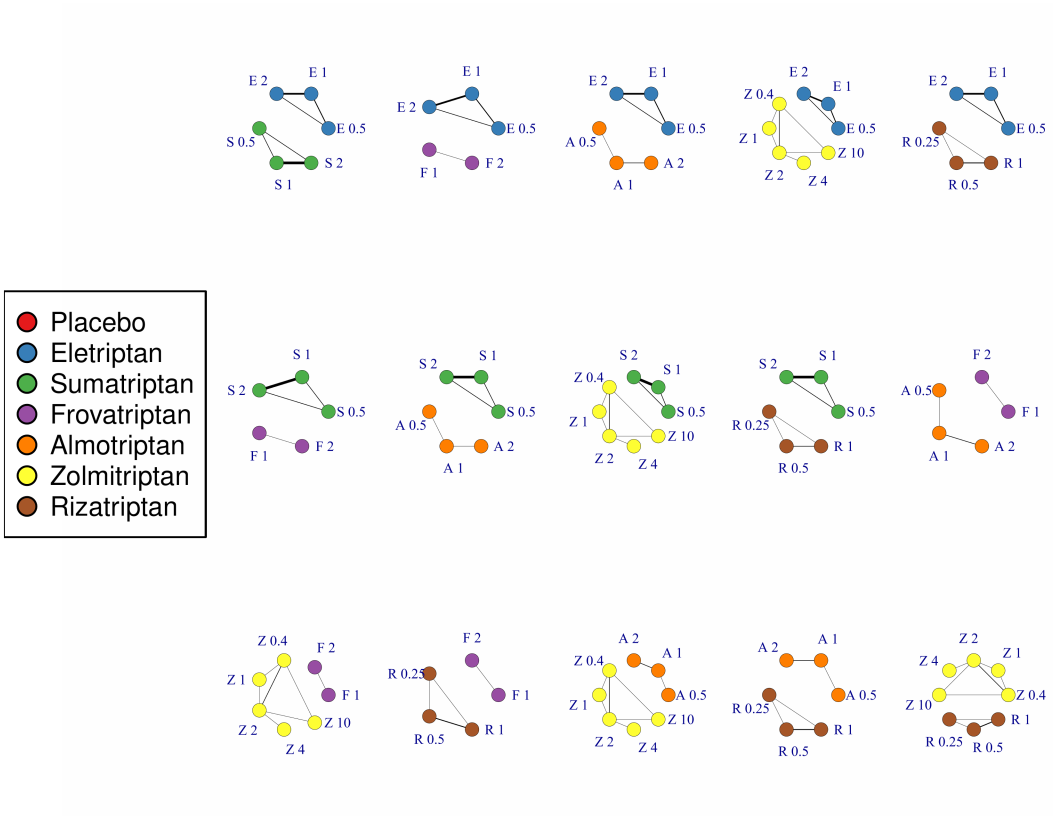

Figure S1: Left panel: Network plots for each of the 15 different disconnected datasets generated in Scenario 2. Each node represents a treatment, named by the first letter of their agent and their dose, standardised to the common dose for each agent. Connecting lines represent direct RCT evidence and their thickness is proportional to the number of studies comparing the connected treatments.

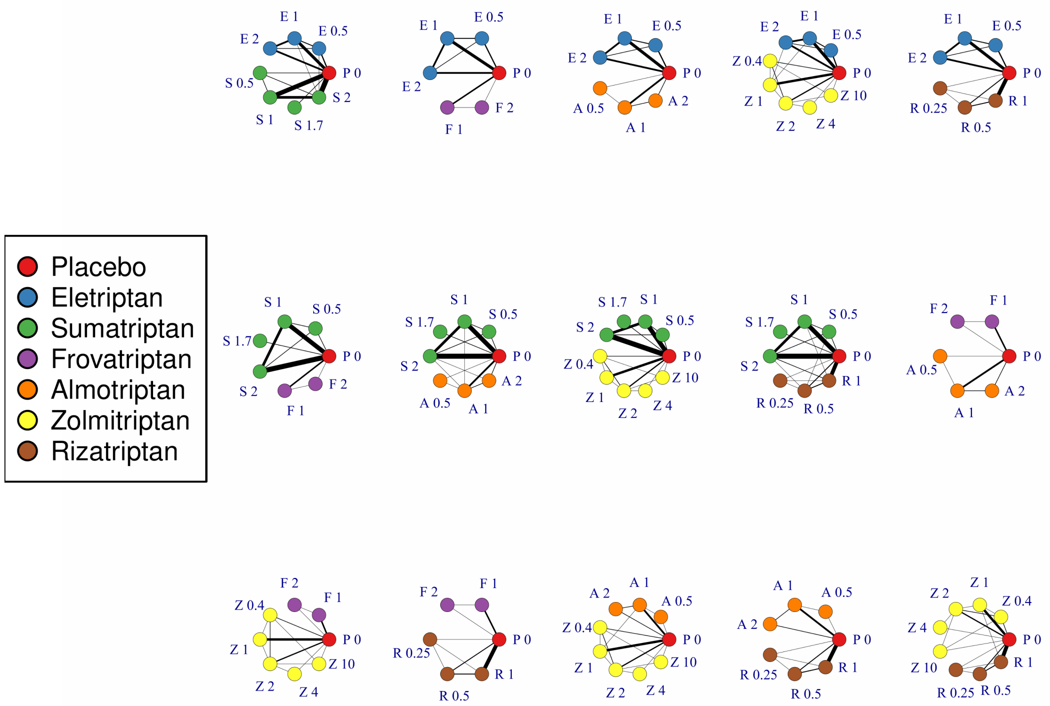

Figure S2: Network plots for each of the 15 different augmented datasets generated in Scenario 2. Each node represents a treatment, named by the first letter of their agent and their dose, standardised to the common dose for each agent. Connecting lines represent direct RCT evidence and their thickness is proportional to the number of studies comparing the connected treatments. The impact of augmentation here is to connect networks that are otherwise disconnected in Figure S1.

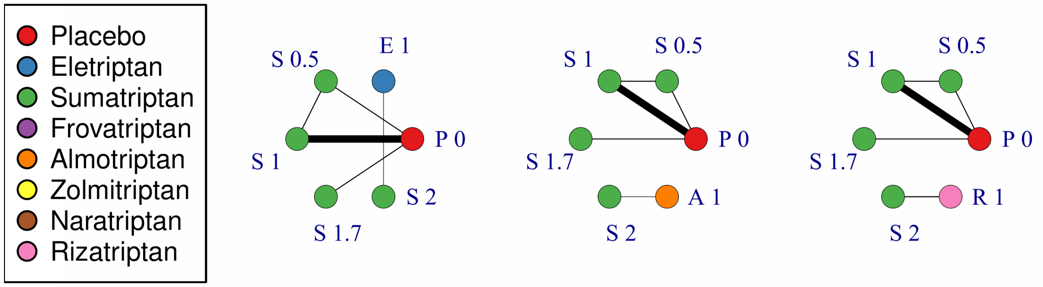

Figure S3: Network plots for each of the 3 different disconnected datasets generated in Scenario 3. Each node represents a treatment, named by the first letter of their agent and their dose, standardised to the common dose for each agent. Connecting lines represent direct RCT evidence and their thickness is proportional to the number of studies comparing the connected treatments.

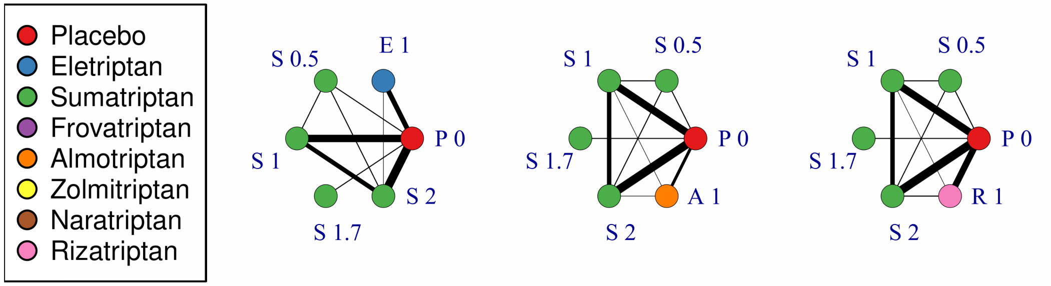

Figure S4: Network plots for each of the 3 different augmented datasets generated in Scenario 3. Each node represents a treatment, named by the first letter of their agent and their dose, standardised to the common dose for each agent. Connecting lines represent direct RCT evidence and their thickness is proportional to the number of studies comparing the connected treatments. The impact of augmentation here is to connect networks that are otherwise disconnected in Figure S3.
